# New insights into the morphology and evolution of the ventral pharynx and jaws in Histriobdellidae (Eunicida, Annelida)

**DOI:** 10.1186/s40851-020-00168-2

**Published:** 2020-11-19

**Authors:** Alexander Tzetlin, Nataliya Budaeva, Elena Vortsepneva, Conrad Helm

**Affiliations:** 1 Department of Invertebrate Zoology, M.V. Lomonosov Moscow State University, Moscow, Russia; 2 Department of Natural History, University Museum of Bergen, University of Bergen, Bergen, Norway; 3 P.P. Shirshov Institute of Oceanology, Russian Academy of Sciences, Moscow, Russia; 4 Animal Evolution and Biodiversity, University of Göttingen, Göttingen, Germany

**Keywords:** Polychaeta, Eunicida, Anatomy, Musculature, Ultrastructure

## Abstract

The jaw apparatus in several annelid families represents a powerful tool for systematic approaches and evolutionary investigations. Nevertheless, for several taxa, this character complex has scarcely been investigated, and complete comparative analyses of all annelid jaws are lacking. In our comprehensive study, we described the fine structure of the jaw apparatus and the ventral pharyngeal organ (VPO) in *Histriobdella homari* – a minute ectocommensal of lobsters putatively belonging to the Eunicida – using different comparative morphological approaches, including SEM, TEM, CLSM and subsequent 3D reconstruction. The *H. homari* jaw apparatus is composed of ventral paired mandibles and dorsal symmetrical maxillae consisting of numerous dental plates, ventral carriers and an unpaired dorsal rod, and the general assemblage and arrangement of the different parts are highly comparable to those of other eunicid families. The jaw ultrastructure of histriobdellids resembles that of the families Dorvilleidae and (juvenile) Onuphidae. Furthermore, our data reveal that in the process of development of the jaw apparatus, the mandibles, maxillae II and unpaired dorsal rod are formed first, and the remaining maxillae and ventral carriers appear later. Notably, the muscular apparatus differs from that in Dorvilleidae and Onuphidae in terms of the number and arrangement of muscle fibers encompassing the jaws – not only because of the very small size of *Histriobdella* but also because histriobdellid maxillary protraction occurs due to straightening of the dorsal rod and thus requires a different muscular scaffold. Based on our investigations, the arrangement of the muscular apparatus of the jaws, the presence of paired ventral carriers and the dorsal rod, and the morphology of the ventral pharyngeal organ represent a histriobdellid autapomorphy. Our datasets form a basis for further comparative analyses to elucidate the evolution of Eunicida and jaw-bearing Annelida.

## Background

Annelids, one of the largest groups of lophotrochozoan animals [1], exhibit a wide variety of lifestyles and body forms [1, 2]. Although recent phylogenomic analyses provide a solid backbone concerning our understanding of character evolution within Annelida, our knowledge for many annelid families is still fragmentary, and evolutionary hypotheses are often highly controversial [1–3]. The reasons for many unresolved questions are the scarcely available morphological and molecular data regarding certain taxa and the lack of fossil records for annelids. The soft body is very poorly and rarely preserved in a fossil state, such that evolutionary conclusions based on fossilized remnants are hardly possible for many annelid taxa [3]. Hence, the bulk of the fossil annelid material is represented by scolecodonts – the elements of the jaw apparatuses of ancient annelids [3]. Nevertheless, complete jaw apparatuses are extremely rare in the fossil record, and the vast majority of fossilized scolecodonts are preserved as separate jaw plates [3, 4]. Within annelids, Eunicida is one of the most prominent jaw-bearing taxa, and numerous analyses of anatomical features of the jaw apparatus have been published thus far. Notably, evolutionary hypotheses regarding the jaw morphology of different eunicidan families, which are always based on complete apparatuses, have scarcely included extinct families [5–8]. However, the number of extinct families considerably exceeds the number of extant families [8]. One reason might be that the correct reconstruction of fossilized complete jaw apparatuses requires detailed knowledge of the morphology, diversity, fine structure and organogenesis of extant annelid jaws, which are still very incomplete for the majority of jaw-bearing taxa. The general morphology of jaws is quite well described in larger and easily recognizable families within the Eunicida [6, 9–13]. Accordingly, the maxillary apparatuses in extant Eunicida can be of four types: ctenognath (comb-like jaws), prionognath (saw-like jaws), symmetrognath (symmetrical jaws), and eulabidognath (pincer-like jaws) (see also Table 1 for further details). Nevertheless, even for those taxa, comprehensive data on ultrastructure, jaw formation and replacement are still very scarce [12, 18]. Additionally, due to a lack of knowledge, interpretations in terms of homologies between the main jaw elements in different eunicidan families are still under discussion [6]. The jaws of the minute Charlie Chaplin worms – Histriobdellidae Claus & Moquin-Tandon, 1884 – are among the poorly understood and understudied examples.

**Table 1:** Comparison of main jaw features and jaw types in different families within the Eunicida

Family	Ref.	Mandibles	Maxillae					Type of jaws
			Symmetry	Number of rows of dental plates	Presence of unidental forceps-like M1	Dorsal carriers	Ventral carriers	
**Dorvilleidae**	[6]	black, unmineralized, connected via ligament or two parts, ventral cutting plate present or absent	symmetrical	2–4	−/+	unpaired (single) carrier-like struсture	–	ctenognath
**Eunicidae**	[6, 14]	calcified partly fused shafts, ventral cutting plate present	asymmetrical	2	+	paired, broad and short	unpaired and delicate	eulabidognath
**Hartmaniellidae**	[6, 15]	black, not fused	subsymmetrical	2	–	paired, moderately long to short	–	symmetrognath
**Histriobdellidae**	this study	black, partly fused with long shafts, cutting plate present	symmetrical	2	–	unpaired, long (rod-like)	paired, short, sliding along mandibular shafts	ctenognath
**Lumbrineridae**	[6, 16]	lightly calcified, completely or partly fused, cutting plate present	symmetrical	2	−/+	paired, moderately long to short	–	symmetrognath
**Oenonidae**	[17]	black, unmineralized, connected via ligament, ventral cutting plate present	symmetrical plate number, plate size can vary between both sides	2	−/+	paired, long	unpaired, long, well sclerotized	prionognath
**Onuphidae**	[6]	calcified partly fused shafts, ventral cutting plate present	asymmetrical	2	+	paired, broad and short	unpaired and delicate	eulabidognath

Histriobdellidae is a scarcely studied family of extant Eunicida with a still-unresolved phylogenetic position [2, 19, 20] and fragmentary knowledge concerning the type of jaw apparatus according to the accepted classification of eunicidan maxillae [6]. The minute histriobdellids are ectosymbionts of marine and freshwater isopod and decapod crustaceans, living commensally in their branchial chambers and on egg masses. The family includes only three genera and 13 described species: the monospecific genera *Histriobdella* Van Beneden, 1858 and *Steineridrilus* Zhang, 2014, as well as the most species-rich taxon, *Stratiodrilus* Menchini, 1900, comprising 11 species [21–24]. The tiny (0.5–1.5 mm long), delicate body of histriobdellids is highly modified with little or no body segmentation and lacks well-defined parapodia or chaetae. The head possesses several tentacular appendages, which are currently referred to as a pair of short palps, three short antennae and a pair of anterior locomotory organs with adhesive glands. Furthermore, histriobdellids have dark, highly specialized mouthparts composed of two groups of jaw elements: mandibles and maxillae [25]. Since the beginning of the twentieth century, Histriobdellidae have been classified as Eunicida based on the presence of these jaws [26–31]. However, their exact placement on the phylogenetic tree is still pending due to the lack of morphological and molecular data.

In their outstanding paper, Jennings and Gelder [25] provided a very detailed description of the jaw morphology of *Histriobdella homari* Van Beneden, 1858, and described the muscular system and the movement of the jaws. They showed that backward movement of the maxillae is ensured by muscle contraction (ventral carrier retractors and dorsal rod flexors), while forward movement of the maxillae is associated with straightening of the dorsal rod.

While the homology of mandibles and maxillary plates (mxI – mxIV) in *Histriobdella* and other Eunicida has not been dubious since the paper by Mesnil and Caullery [27], the question about the homologues of the histriobdellid dorsal rod and ventral carriers (terminology sensu [25]) in other eunicidan families remains unresolved. Although Tzetlin [31] suggested that Histriobdellidae possess two-rowed, comb-shaped (ctenognath) maxillae, Paxton [6] did not include Histriobdellidae in her analysis of the recent and fossil eunicidan jaws and referred to the jaw shape as unlike any of the previously recognized types of eunicidan jaws. Ultrastructural data of the jaws and the muscles of the ventral pharyngeal organ of histriobdellids are still fragmentary [30], and no data on the development of histriobdellid mouth parts have been published.

Hence, the present study aims to broaden our knowledge concerning detailed features of the histriobdellid jaw apparatus. Using a comparative set of morphological methods, we investigated the fine morphology and ultrastructure of the ventral pharyngeal organ and jaw apparatus in *Histriobdella homari* to place the histriobdellid jaw apparatus within the accepted classification of eunicidan jaws*.* Such comprehensive analyses and resulting hypotheses concerning putative homologies between different eunicidan jaw apparatuses will help elucidate the evolution of the jaw-bearing Eunicida. Furthermore, detailed analyses of underinvestigated annelid groups will deepen our understanding of Annelida character evolution scenarios in general.

## Material and methods

### Specimen collection

Specimens of *Histriobdella homari* were collected from their hosts, *Homarus gammarus* (Linnaeus, 1758), which were obtained from the central fish market in Bergen (Norway) in December 2016 and June 2017 and originated from the northern Atlantic. The adult and juvenile individuals were washed from their hosts using 7% MgCl_2_ in seawater (1:1). Histriobdellid eggs attached to the host were collected using dissecting needles.

### Immunohistochemistry

Anatomical details of adults and developmental stages of *H. homari* were investigated in whole animal preparations using standard immunohistochemical staining protocols and phalloidin-rhodamine as a muscular marker. The staining was carried out using 15–25 specimens of each stage. The individuals were relaxed in a 7% MgCl_2_ seawater (1:1) solution and subsequently fixed in 4% paraformaldehyde (PFA) in 1x phosphate-buffered saline with Tween (PTW = 1x PBS: 0.05 M PB/0.3 M NaCl/0.1% Tween 20, pH 7,4) for 2 h at room temperature. After fixation, the specimens were stored in PTW containing 0,005% NaN_3_ until usage at 4 °C.

For investigations, specimens were rinsed 2x for 5 min in PTW at RT (room temperature) and transferred into 10 μg proteinase K/ml PTW for 10 min. After 2 short rinses in glycine (2 mg glycine/ml PTW) and 3 5-min washes in PTW, the specimens were refixed using 4% PFA in PTW containing 0.1% Tween for 20 min at RT. Subsequently, the samples were rinsed 2x for 5 min in PTW, rinsed 2x for 5 min in THT (0.1 M Tris-Cl, 0.1% Tween, pH 8,5) and incubated with rhodamine-labeled phalloidin in THT (Invitrogen, Carlsbad, CA; 5 μl methanolic stock solution in 500 μl THT) overnight at 4 °C.

Subsequently, the specimens were dehydrated in an ascending isopropanol series, cleared using Murray’s clear (benzyl alcohol and benzyl benzoate, 1:2) and embedded between two cover slips using DPX mounting medium (Merck, Darmstadt, Germany). The specimens were analyzed with a Leica TCS SP5 confocal laser-scanning microscope (Leica Microsystems, Wetzlar, Germany). The confocal image stacks were processed with Leica AS AF v2.3.5 (Leica Microsystems) and Imaris 9.3 (Bitplane AG, Zurich, Switzerland).

### Electron microscopy – SEM & TEM

For electron microscopy (EM), the specimens were fixed in a 2.5% glutaraldehyde solution buffered in 0.05 M phosphate and 0.3 M saline (PBS) (pH 7.2) at 4 °C for 1 h and kept in the same buffer. For scanning electron microscopy (SEM), the specimens were postfixed in 1% OsO4 buffered in 0.05 M phosphate and 0.3 M saline at 4 °C for 30 min and then immediately dehydrated in an ascending ethanol series. The samples dehydrated in 100% ethanol were transferred into microporous specimen capsules (Electron Microscopy Sciences, Hatfield, USA) for subsequent critical-point drying. The jaws were freed from the surrounding tissue using proteinase K in PTW (overnight at RT), rinsed in distilled water, transferred to coverslips, and air dried. Animals were mounted on conductive carbon adhesive tabs on pin stubs (Electron Microscopy Sciences Hatfield, USA). Both the jaws and the specimens were subsequently sputter-coated with 40% gold:60% palladium (Polaron SC502 Sputter Coater) and examined under a Zeiss SUPRA 55VP field emission scanning electron microscope. Image acquisition was performed using a secondary electron detector at a 3 kV accelerating voltage with a 30 μm aperture. The final images were processed using Adobe (San Jose, CA, USA) Photoshop CC and Illustrator CC.

For transmission electron microscopy (TEM) and the preparation of semithin section series, the fixed specimens were dehydrated in an ascending ethanol series, transferred to acetone and embedded in Spurr’s resin. The series of semithin and ultrathin sections were obtained with DuPont MT 5000 and Leica EM UC6 ultramicrotomes. The semithin sections were subsequently stained with 1% toluidine blue and 1% methylene blue in 1% sodium tetraborate. The ultrathin sections were stained with uranyl acetate (1%, 40 min, 35 °C) and lead citrate (0,4%, 10 min, room temperature) and examined with a Jeol JEM 1011 transmission electron microscope. 3-D reconstructions were performed from a series of semithin sections. Based on the image stacks that were aligned into stacks using the software AMIRA 5.2.2 (Amira Visaging GmbH, Germany), the ventral pharyngeal organ and its musculature were reconstructed using the software Imaris 7.0.0 (Bitplane AG, Zurich, Switzerland).

## Results

### General structure of the mouthparts

The description of the general anatomy of the stomodeum in *H. homari* is based on the terminology suggested by Tzetlin and Purschke [13, 18]. The detailed description of the jaw structures follows the terminology suggested by Jennings & Gelder [25].

**Fig. 1:**
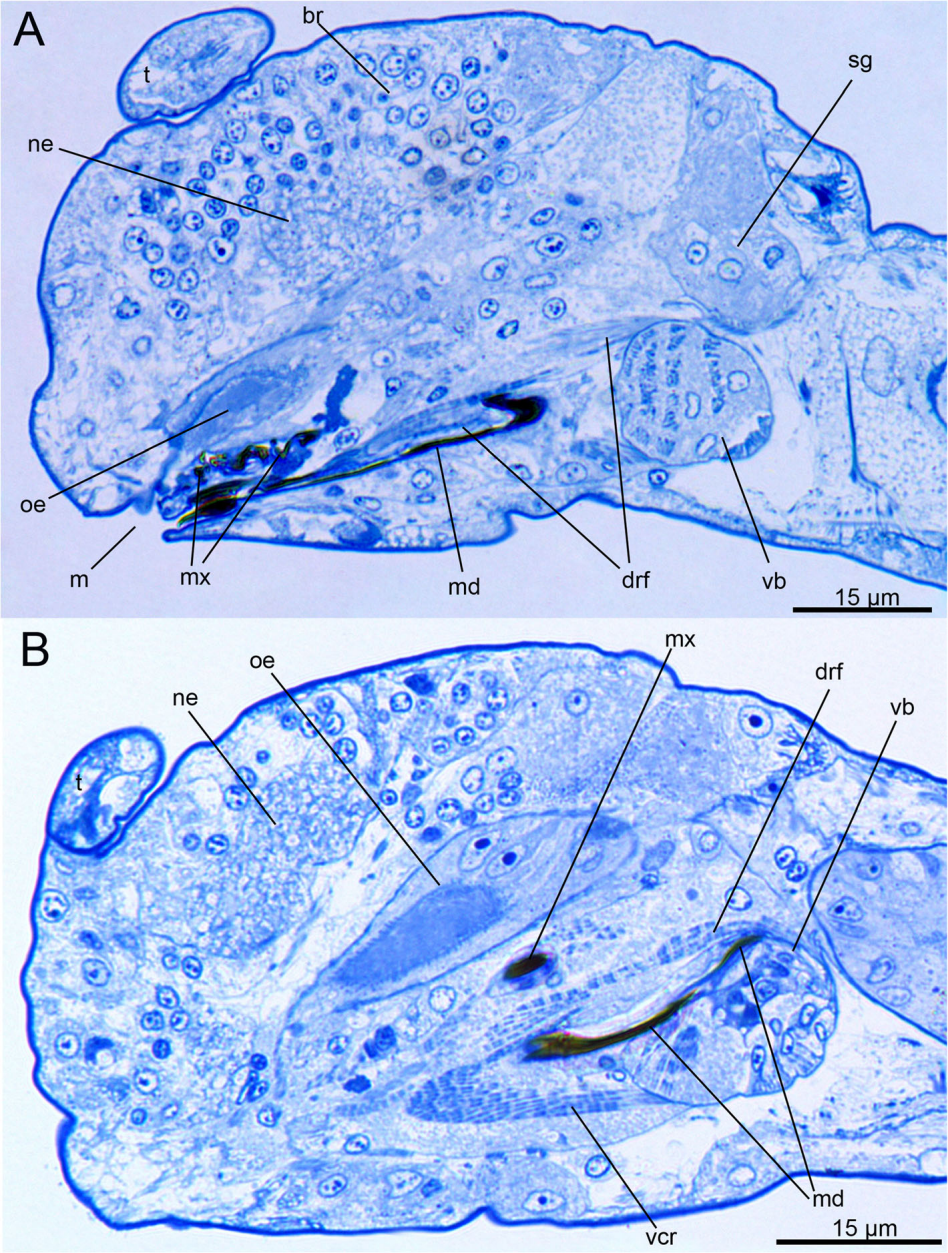
General morphology of the anterior end of *Histriobdella homari*. Semithin sections, light microscopic images. **a.** Parasagittal section through the mouth region. **b.** Parasagittal section through the muscular apparatus of the ventral pharyngeal organ and jaws. br, somata of the brain; drf, dorsal rod flexor; m, mouth; md, mandibula; mx, maxillae; ne, brain neuropil; oe, esophagus; t, tentacle; sg, salivary gland; t, tentacles; vb, ventral muscle bulb; vcr, ventral carrier retractor

**Fig. 2:**
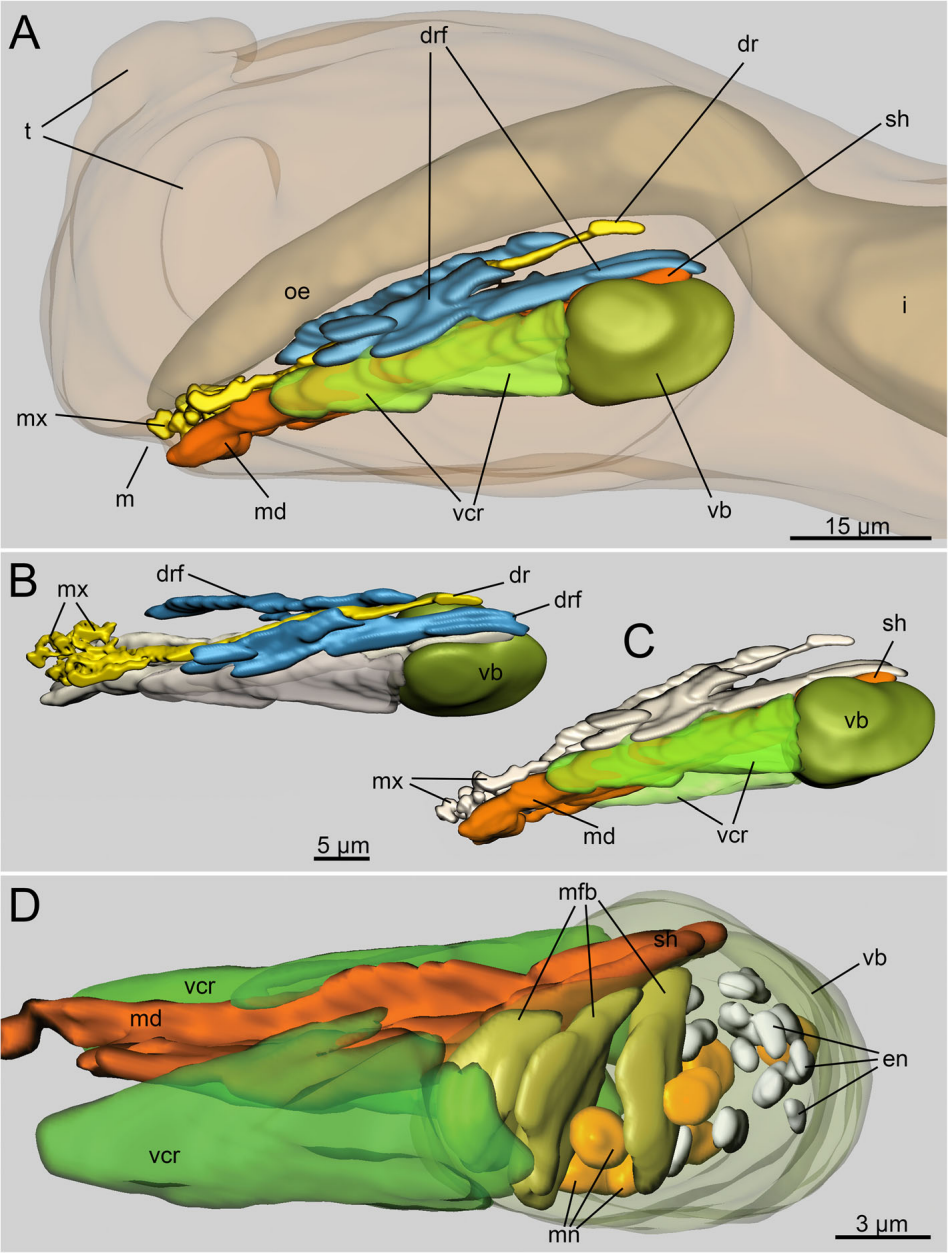
Three-dimensional (3D) reconstruction of the jaw apparatus of *Histriobdella homari*. The main jaw elements are indicated in different colors: maxillae and dorsal rod – yellow; dorsal rod flexors – blue; mandibles and mandibular shaft – orange; ventral carrier retractors – light green; ventral muscle bulb – olive; epithelial nuclei – gray; muscular nuclei – light orange. **a.** General view of the head with the intestine, maxillae, mandibles and muscles. Lateral view. **b.** Ventral pharyngeal organ (VPO) and jaws, dorsolateral view. **c.** Ventral pharyngeal organ and jaws, ventrolateral view. **d.** Detailed reconstruction of the VPO. Lateral view. Drf, dorsal rod flexor; dr, dorsal rod; en, epithelial cell nuclei in the ventral muscle bulb; i, intestine; m, mouth; md, mandible; mfb, transversal muscle fibers of the ventral bulb; mn, muscular nuclei of the ventral bulb; mx, maxillae; oe, esophagus; sh, mandibular shaft; t, tentacle; vb, ventral muscle bulb; vcr, ventral carrier retractor

In *H. homari,* the jaws are relatively large in comparison to the body size of the adult worms (approximately 1.3–1.5 mm for adult specimens investigated in this study). The entire ventral pharyngeal organ (VPO) in the respective specimens ranges from 70 to 80 μm in length and approximately 40–45 μm in width (Fig. 1a, b; Figs. 2a).

The esophagus originates from the posterodorsal part of the oral cavity and bends around the oral cavity dorsally before running towards the intestine (Figs. 1a; Figs. 2a). The epithelium of the oral cavity as well as the epithelium of the VPO is coated with a well-defined cuticle but does not exhibit cilia. In contrast, the epithelia of the esophagus and the intestine show dense and long cilia, which fill the entire lumen of the respective structures.

The VPO of *H. homari* bears prominent jaws (mandibles and maxillae), which are located directly behind the mouth opening in the oral cavity (Figs. 1a, b). The scraping edge (margin) of the mandibles, which bears teeth, is everted from the oral cavity when the mouth is open in adult specimens (Fig. 1a).

**Fig. 3:**
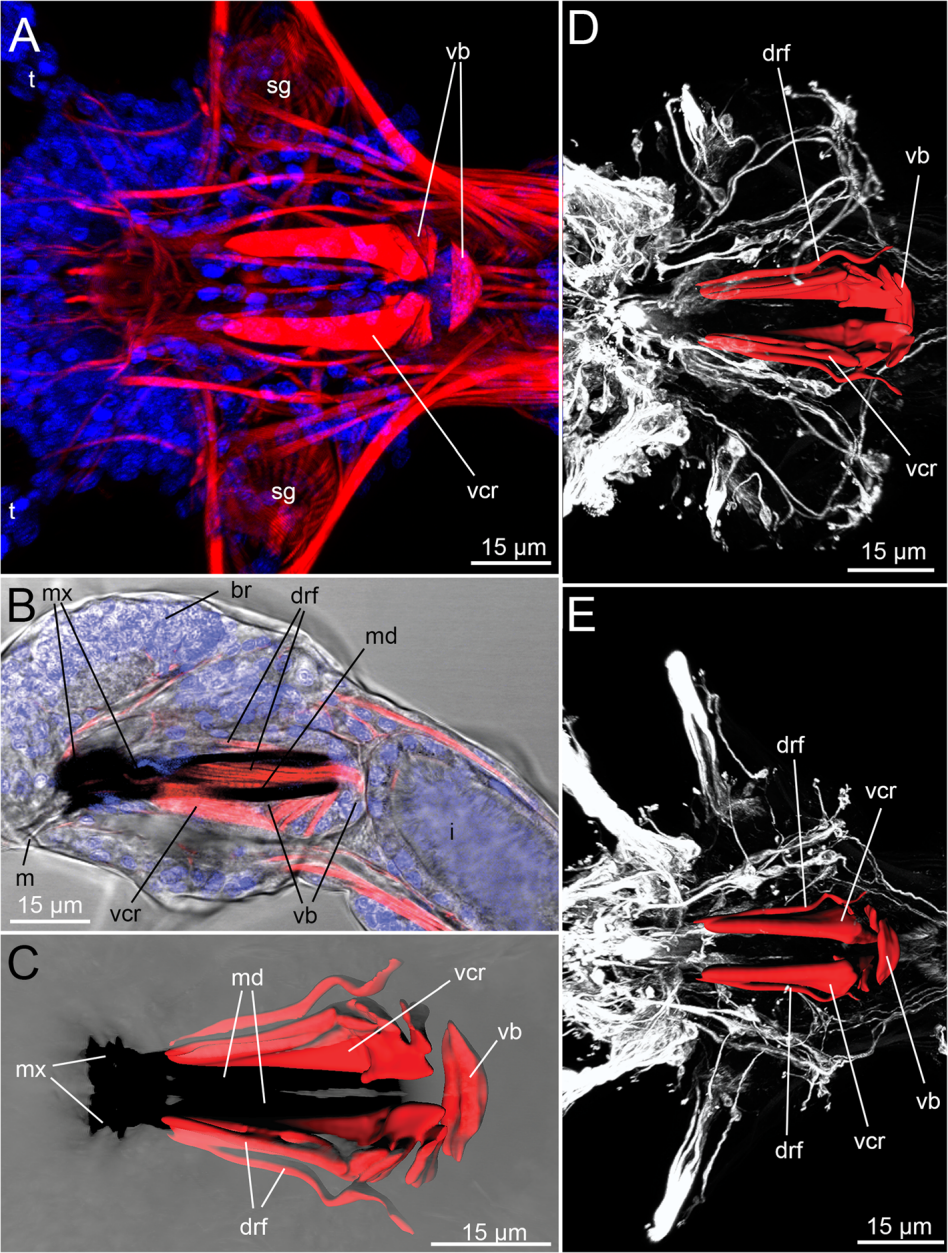
Muscular apparatus serving the jaws of *Histriobdella homari*, confocal maximum projections. Jaws – black; nuclei – blue; musculature – red; tubulinergic nervous system – white. **a.** General view of the muscular apparatus. Ventral view. **b.** Lateral view of the anterior end, transmission light microscopy and cLSM. **c.** 3D reconstruction of the buccal muscles of the ventral pharyngeal organ. **d.** The buccal complex with the complete set of muscle elements of the anterior part of the body. Dorsal view. **e.** Ventral view of the buccal complex with the complete set of muscle elements of the anterior part of the body. br, brain; drf, dorsal rod flexor; sg, salivary gland; m, mouth; md, mandibula; mx, maxillae; t, tentacle; vb, ventral muscle bulb; vcr, ventral carrier retractor

**Fig. 4:**
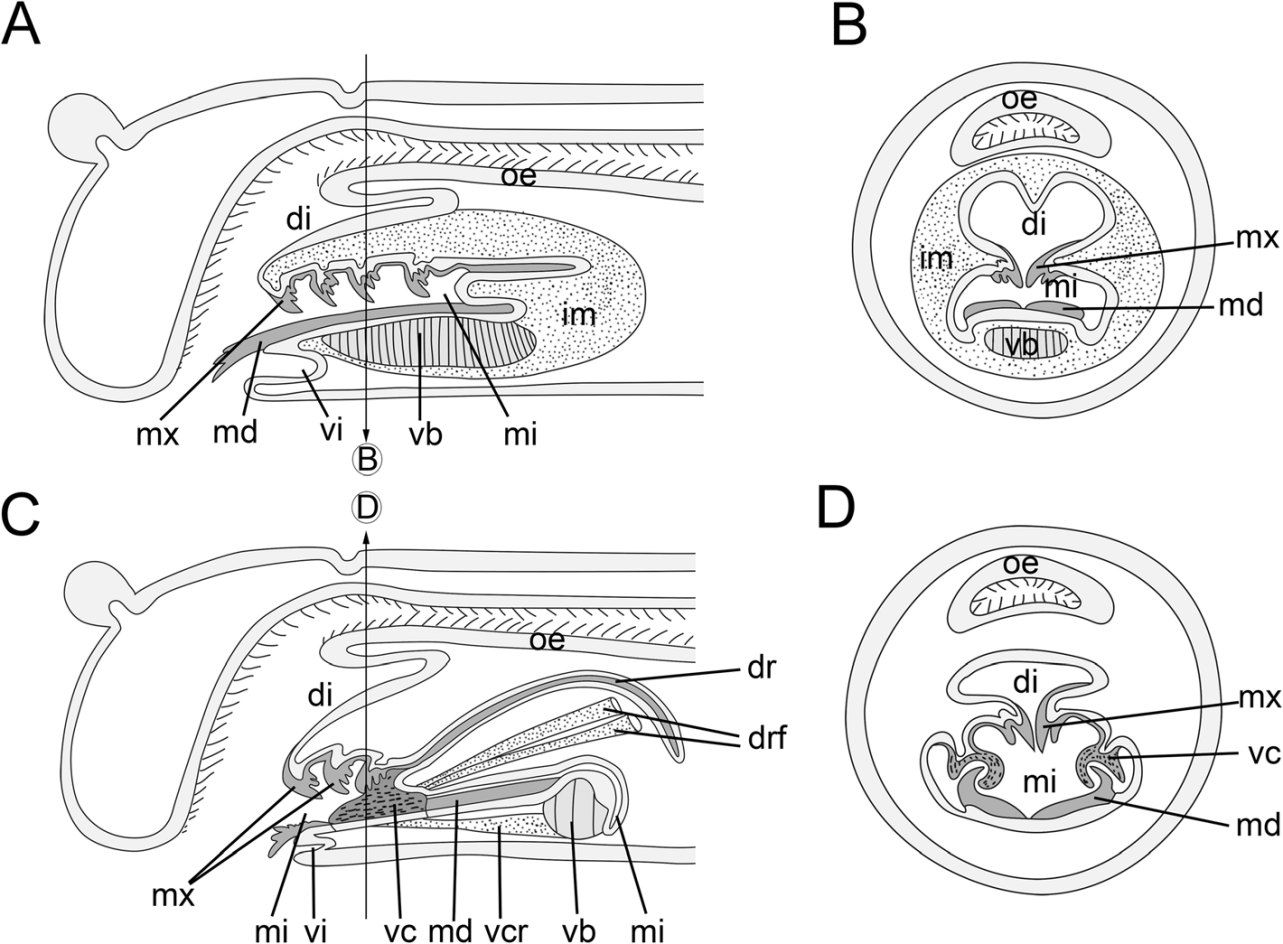
Comparative schemes of the pharyngeal apparatuses in Dorvilleidae and *Histriobdella homari*. Arrows in (**a**) and (**c**) indicate the position of the transversal section. **a.** Dorvilleidae (*Ophryotrocha* and *Protodorvillea*); lateral view. **b.** Dorvilleidae (*Ophryotrocha* and *Protodorvillea*); cross-section. **c.** *Histriobdella*; lateral view. **d.** *Histriobdella*; cross-section. The schematic representation is modified from [9]. di, dorsal invagination; dr, dorsal rod; drf, dorsal rod flexor; im, investing muscles; md, mandibles; mi, median invagination; mx, maxillae; oe, esophagus; vb, ventral muscle bulb; vc, ventral carriers; vcr, ventral carrier retractor; vi, ventral invagination

**Fig. 5:**
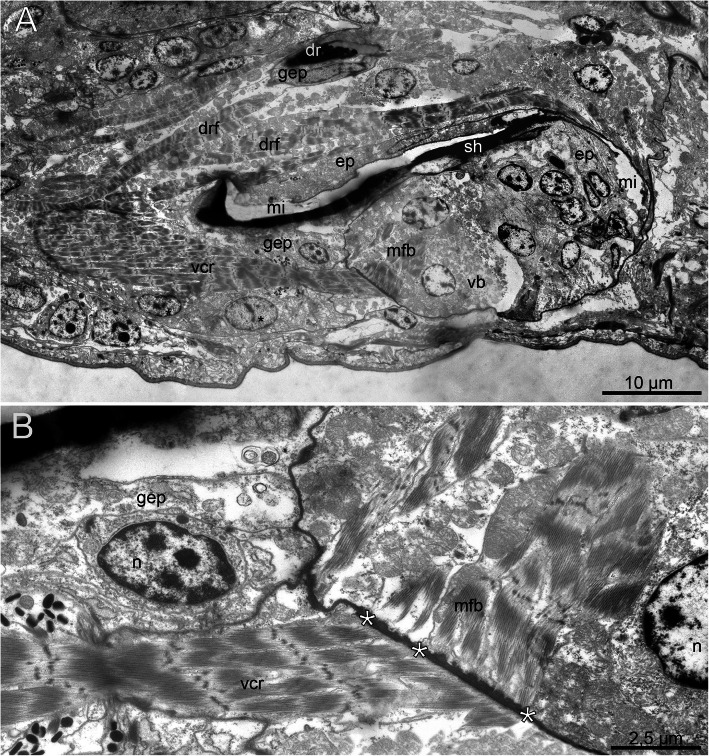
Fine structure of the ventral pharyngeal organ (VPO) and jaws of *Histriobdella homari*. Parasagittal section, TEM images. **a.** General overview of the VPO. Black asterisks mark nuclei of the gnathoepithelial cells. **b.** Сontact zone between muscle fibers of the ventral bulb and the ventral carrier retractor. White asterisks mark the zone with dense hemidesmosomes. dr, dorsal rod; drf, dorsal rod flexor; ep, epithelium of the median VPO invagination; gep, gnathoepithelial cell; mfb, muscle cell of the ventral bulb; mi, median VPO invagination; n, nucleus; sh, mandibular shaft; vb, ventral muscle bulb; vcr, ventral carrier retractor

In addition to the jaw plates, the VPO contains a well-defined gnathoepithelium and very few large muscle cells for jaw movements and manipulations (Fig. 2a-d; Fig. 3a-c). Interestingly, the VPO does not exhibit an obvious connection to the muscles of the body wall (Fig. 1a-b; Fig. 2a-d; Fig. 3a-e). Furthermore, *H. homari* does not possess a complete muscle capsule covering the VPO (Fig. 3a-e; Fig. 4c, Fig. 5a). Its muscle apparatus solely consists of a few muscle cells: retractors of the ventral carriers and flexors of the unpaired dorsal rod (Fig. 1a; Fig. 2a-d; Fig. 3c; Fig. 4c).

**Fig. 6:**
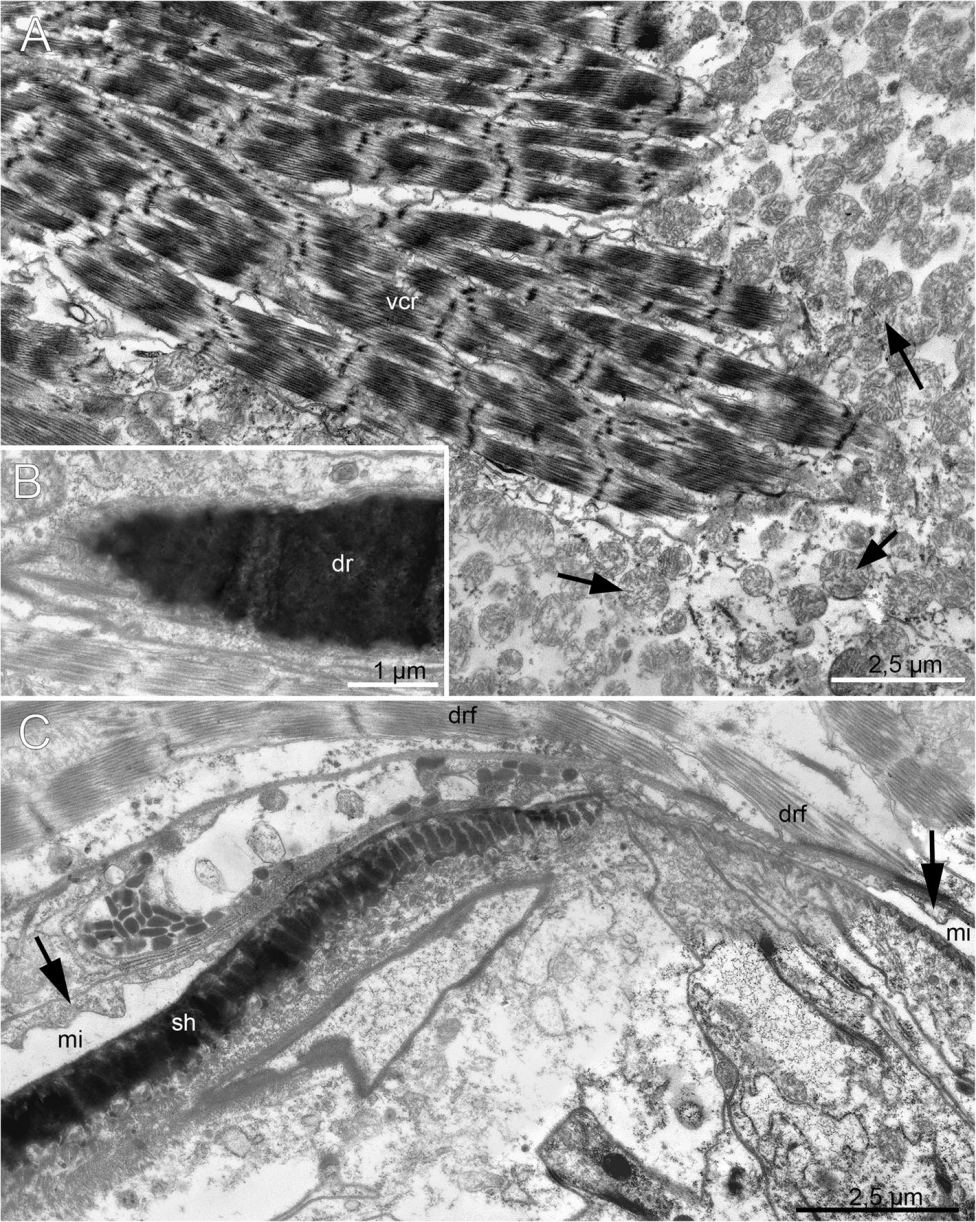
Fine structure of the ventral pharyngeal organ (VPO) of *Histriobdella homari*. Parasagittal section, TEM images. **a.** Ultrastructure of the ventral carrier retractor. Arrows indicate the position of mitochondria. **b.** Fine structure of the dorsal rod. **c.** Structure of the posterior zone of the median invagination. Arrows indicate very thin pharyngeal epithelial cells. dr, dorsal rod; drf, dorsal rod flexor; mi, median VPO invagination; sh, mandibular shaft; vcr, ventral carrier retractor

In total, three invaginations can be observed along the entire structure of the VPO. The ventral invagination is located just behind the mouth opening, while the dorsal invagination is found between the VPO and the esophagus (Fig. 1a; Fig. 4c-d). The ventral and dorsal invaginations are shallow and much smaller than the median invagination (Fig. 4c-d). The dorsal invagination runs along the entire VPO structure and forms a narrow space between the mandibles (mandibular shafts), the maxillae and the dorsal rod. The median invagination curves dorsally around the muscular bulb and ends posteriorly to the dorsal invagination bulb (Fig. 4c; Fig. 5a). All three invaginations are outlined by an epithelium (Fig. 5a; Fig. 6b). The elements of the maxillary apparatus are situated on the surface of the median invagination in the frontal (extended maxillae) or anterior (retracted maxillae) part of the VPO (Fig. 4c, d). The mandibles are located on the ventral side of the median invaginations. The ends of the mandibular shafts rest on the frontal and dorsal edges of the ventral muscle bulb (Fig. 2a-d; Fig. 4c).

### Muscles of the ventral pharyngeal organ (VPO)

The VPO muscle complex consists of several groups of relatively large muscle cells. The composition of these cells is surprisingly constant in comparison with that of other eunicidans. Thus, paired ventral carrier retractors, a few pairs of dorsal rod flexors and a distinct ventral muscle bulb are present (Fig. 2a-d; Fig. 3a-e; Fig. 4c). The ventral carrier retractors (two cells) are large cells with oblique muscle fibers and numerous mitochondria (0.3–0.8 μm) located in the posterior lateral, more voluminous part of the cells (Fig. 2a-d; Fig. 3c,e; Fig. 4c; Fig. 5a-b; Fig. 6a). In their anterior part, these cells contact the gnathoepithelium cells of the ventral carrier. The posterior part contacts the ventral muscle bulb cells (Figs. 4C; 5A, B). The ventral bulb itself consists of large muscle cells with oblique striated myofilaments oriented transversally (Figs. 2d; Fig. 3c; Fig. 5a).

According to our data, there are just three bulb muscle cells arranged in a row, one after another (Fig. 2d). These cells are of the platimyarian type – with a flat zone of myofilaments arranged in one layer and a more voluminous cytoplasmic posterior part containing mitochondria and a nucleus (Fig. 5a).

The muscle fibers of the dorsal rod flexors are represented by 8–9 pairs (left and right) of muscle cells (Figs. 2a; Fig. 3c-e; Fig. 4c; Fig. 5a; Fig. 6c). These long and narrow cells appear from the gnathoepithelial cells surrounding the anterior part of the dorsal rod, run along the dorsal side of the median invagination and rest on the epithelium of the posterior-most part of the median invagination behind the muscle bulb (Fig. 5a; Fig. 6c). These muscle cells likely represent cells of the circomyarian type - with cell bodies filled with mitochondria and nuclei located posteriorly.

### Composition of the jaws

**Fig. 7:**
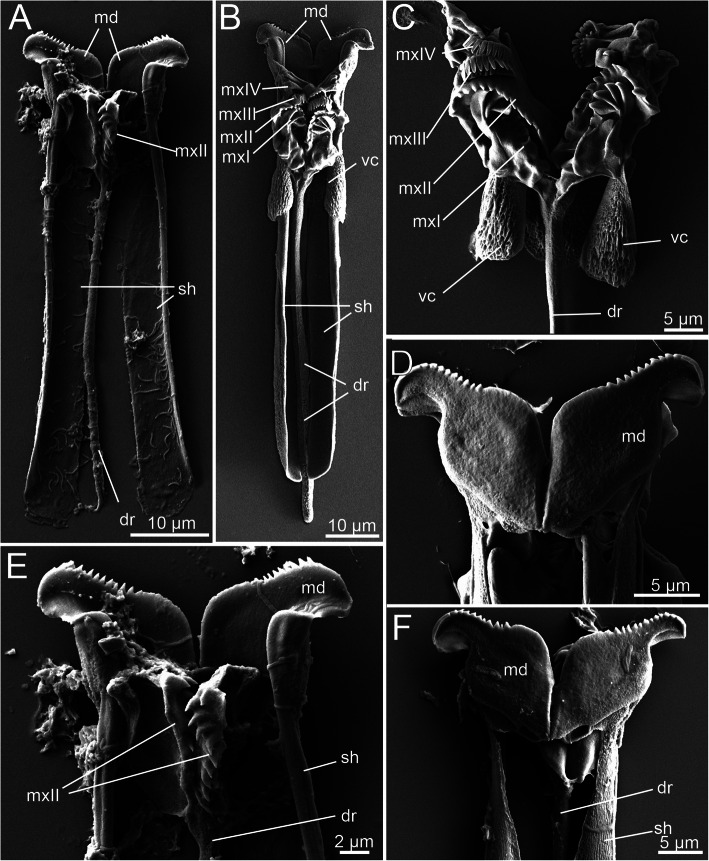
General morphology of jaw components in *Histriobdella homari*. SEM images. All jaws are shown in dorsal view, except for (D) and (F), which are shown ventrally. **a.** Juvenile jaw apparatus with the main components. **b.** Adult jaw apparatus. **c.** Detailed view of the adult maxillae. **d.** Anterior end of the adult mandibles. **e.** Anterior part of the juvenile jaw apparatus. **f.** Anterior half of the juvenile jaw apparatus. dr, dorsal rod; md, mandible; mxI – mxIV, maxillary plates I – IV; sh, mandibular shaft; vc, ventral carriers

The jaws in histriobdellids – as well as in other eunicids – comprise ventral mandibles and dorsal maxillae (Figs. 1a, b; Fig. 4c-d; Fig. 7b). Every mandible thereby exhibits a well-defined anterior scraping part (mandibular plate), approximately 12–13 μm wide, and a long mandibular shaft embedding the entire structure within the VPO (Fig. 7b). The length of the mandibular shaft is approximately 70 μm, so the total length of the mandibles in adult specimens reaches approximately 80 μm.

The paired mandibles are fused along the mandibular plates and attached to each other in the median part of the mandibular shafts (Figs. 7b, d-f). The frontal margin of the scraping part of each mandible bears approximately 10 denticles, which are 0.5 μm in height (Figs. 7b, d-f). The posterior portion of each mandibular shaft appears flattened and elongated and rests on the posterior part of the pharyngeal muscle bulb in living animals (Fig. 7 b). The mandibular shafts are flattened and curved when observed in transverse sections, approximately 2.5 μm wide in the anterior part and approximately 5–6 μm wide in the posterior part (Figs. 7b, d-f).

**Fig. 8:**
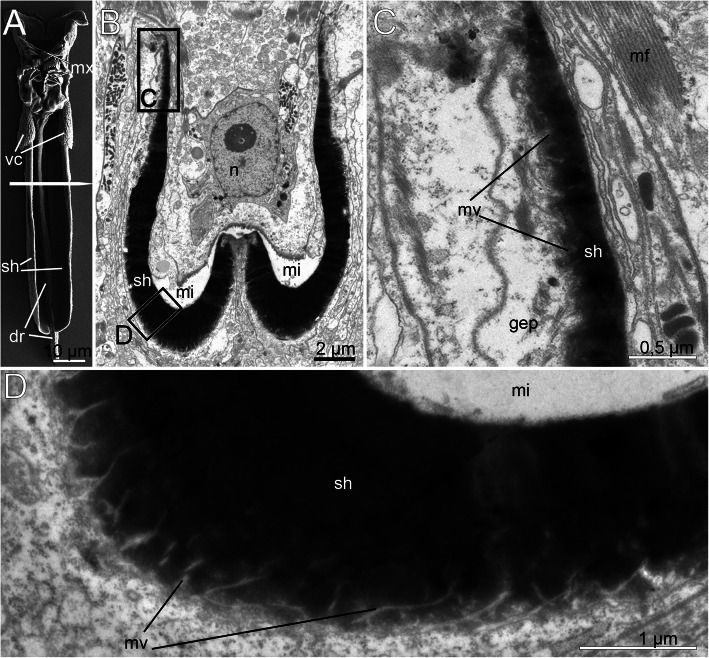
Fine structure of the adult mandibles of *Histriobdella homari*. **a.** Jaws with a maxillary apparatus and mandibles, SEM image of the entire jaw apparatus. The white line indicates the position of the transversal ultrathin sections. **b.** Ultrastructure of the mandibular shaft; transversal section. The black squares correspond to TEM images shown in (**c**) and (**d**). **c**. *Close*-up of the mandibular margin. **d**. Ultrastructure of the median mandible. dr, dorsal rod; gep, gnathoepithelial cell; mi, median VPO invagination; mv, zone with microvilli; n, nucleus; sh, mandibular shaft

The mandibles are the most prominent components of the jaw apparatus in *H. homari*. The thickness of the mandibular plates and the mandibular shafts reaches 2.5 μm, whereas the margins of the mandibular complex are much thinner (Figs. 8a-d). At the margin of the mandibular plate, the black electron-dense layer becomes thinner. Here, it gradually transforms into a thin layer of the epithelial epicuticle (Fig. 8c). Interestingly, the mandibular plates are almost monolithic and show less electron-dense structures. They are formed from fused granules with a diameter of 0.05 μm, which can be clearly seen at the periphery of the plate in the TEM micrographs (Figs. 8b-d).

The mandibles are even present and visible in late embryos and juveniles. The first elements of the jaw apparatus that appear in the *Histriobdella* embryos are the two lines of denticles of the mandibular cutting plates. The juveniles – which are still inside the egg envelope and do not feed – have already fully developed mandibles that exhibit the same size and shape as those of adult free-living and feeding individuals (Fig. 7a).

Notably, the situation differs for the second main component of the histriobdellid jaw apparatus – the maxillae. Early juveniles of *H. homari*, which are still inside the egg envelope and already show well-developed mandibles, exhibit only parts of the maxillary apparatus. Hence, only the incompletely developed plates mxII and the dorsal rod are present at this stage of development (Figs. 7a, e). Additional maxillary plates (mxI, mxIII and mxIV), which are observable in adult worms, are not yet developed (see Fig. 7b for comparison). Furthermore, these early stages do not possess ventral carriers, which are typical for the adult maxillae (Figs. 7b-c). Nevertheless, juvenile individuals emerging from egg membranes already have a fully developed jaw apparatus comparable to that in adults. The general arrangement of the jaw apparatus does not change between juveniles and adults (Figs. 7a-b).

In late juveniles and adults, the fully developed maxillary apparatus consists of four pairs of maxillary plates equipped with a special supportive apparatus, namely, the paired ventral carriers and the unpaired dorsal rod (Figs. 7b, c). Between maxillary plates II and III, few additional jaw elements of an indefinite shape exist (Fig. 7c). The cuticle of these additional jaw elements is relatively thin and elastic. Interestingly, the shape of these slightly armored elements differs among the observed specimens.

Maxillary plate I (mxI) bears four large teeth (up to 3 μm in height). The tooth located close to the basal part of the plate is the smallest (Fig. 7c). The length of mxI reaches 10–12 μm. The basal part of mxI is situated between the base of the dorsal rod and the place where the maxillary apparatus contacts the ventral carriers. The basal part of the maxillae reaches 1.5 μm in thickness, while the distal part can be less than 0,4 μm thick. Maxillary plate II (mxII) represents the densest and largest of all maxillary plates. It bears 11–12 teeth along the anterior margin. The teeth are smaller than those of mxI (not more than 2 μm in height). Maxillary plates III (mxIII) and IV (mxIV) are smaller in size and bear only one row of narrow and relatively tall teeth, up to 2–2.5 μm in height (Fig. 7c).

**Fig. 9:**
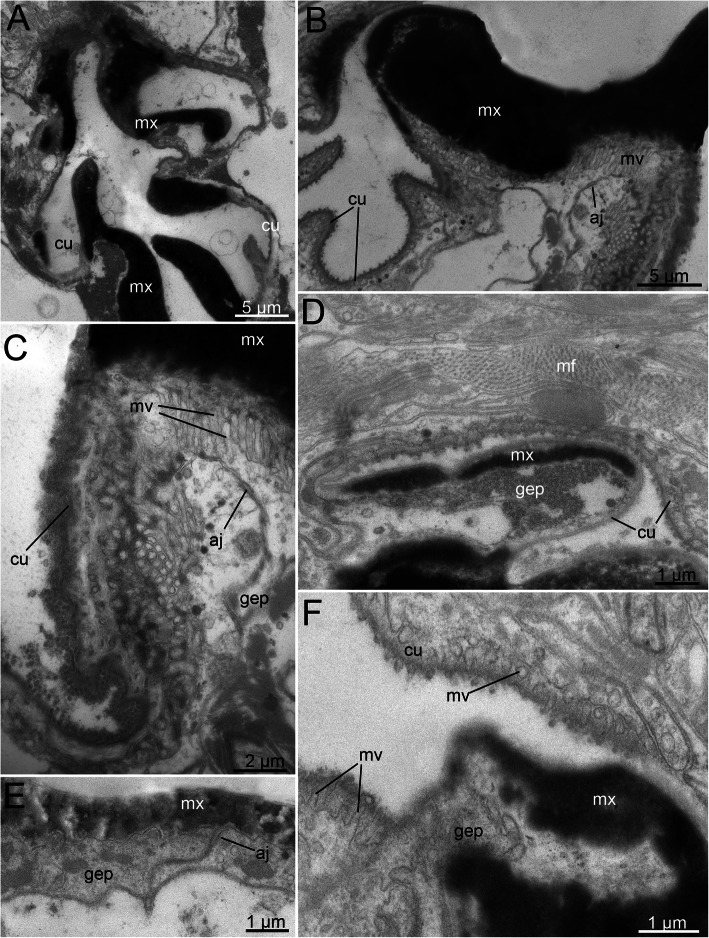
Fine structure of the adult (**a-c**) and juvenile (**d-f**) maxillae of *Histriobdella homari*. TEM images. **a.** Frontal section through the maxillae. **b.** Frontal maxillae, higher magnification. **c.** Transition zone between the solid maxilla and pharyngeal cuticle. **d-f.** Fully formed juvenile maxillae and transitional zone between the maxilla and pharyngeal epithelium. **e.** Fragment of the newly formed juvenile maxilla. aj, adherens junction; cu, pharyngeal cuticle; gep, gnathoepithelial cell; mf, myofilament; mv, microvilli; mx, maxillae

The maximal thickness of the maxillae reaches 0.5–1.0 μm (Fig. 9a-c). The maxillae are formed of highly pigmented and electron-dense material, which at the stage of formation represents a conglomerate of electron-dense granules approximately 0.1–0.2 μm in diameter (Fig. 9b-f). Subsequently, these granules transform into an unstructured electron-dense mass. Notably, the maxillae are positioned on top of the gnathoepithelial cells, which form solid plates in combination with the electron-dense matrix (Figs. 9b-f). In the distal parts of the maxillae, distinct gnathoblasts are present. The gnathoblasts appear to be in a degenerated state: the cytoplasm appears empty and filled with gray granules with a diameter of 0.005–0.01 μm (Figs. 9a, d). In the more basal parts of the maxillae, the gnathoepithelial cells contain mitochondria and a nucleus. Furthermore, the structure of the cytoplasm of the gnathoblasts in these areas indicates normal cellular activity (Fig. 9b-c, e-f).

In the marginal areas of the maxillary plates, it is obvious that the solid, hard body of the maxillae represents a rapidly growing epicuticular part of the cuticle. The layer of living microvilli is preserved underneath (Figs. 9 a-f). A layer of unstructured gray material is situated between the solid maxillary plate and the microvilli of the gnathoepithelial cells (Fig. 9c).

**Fig. 10:**
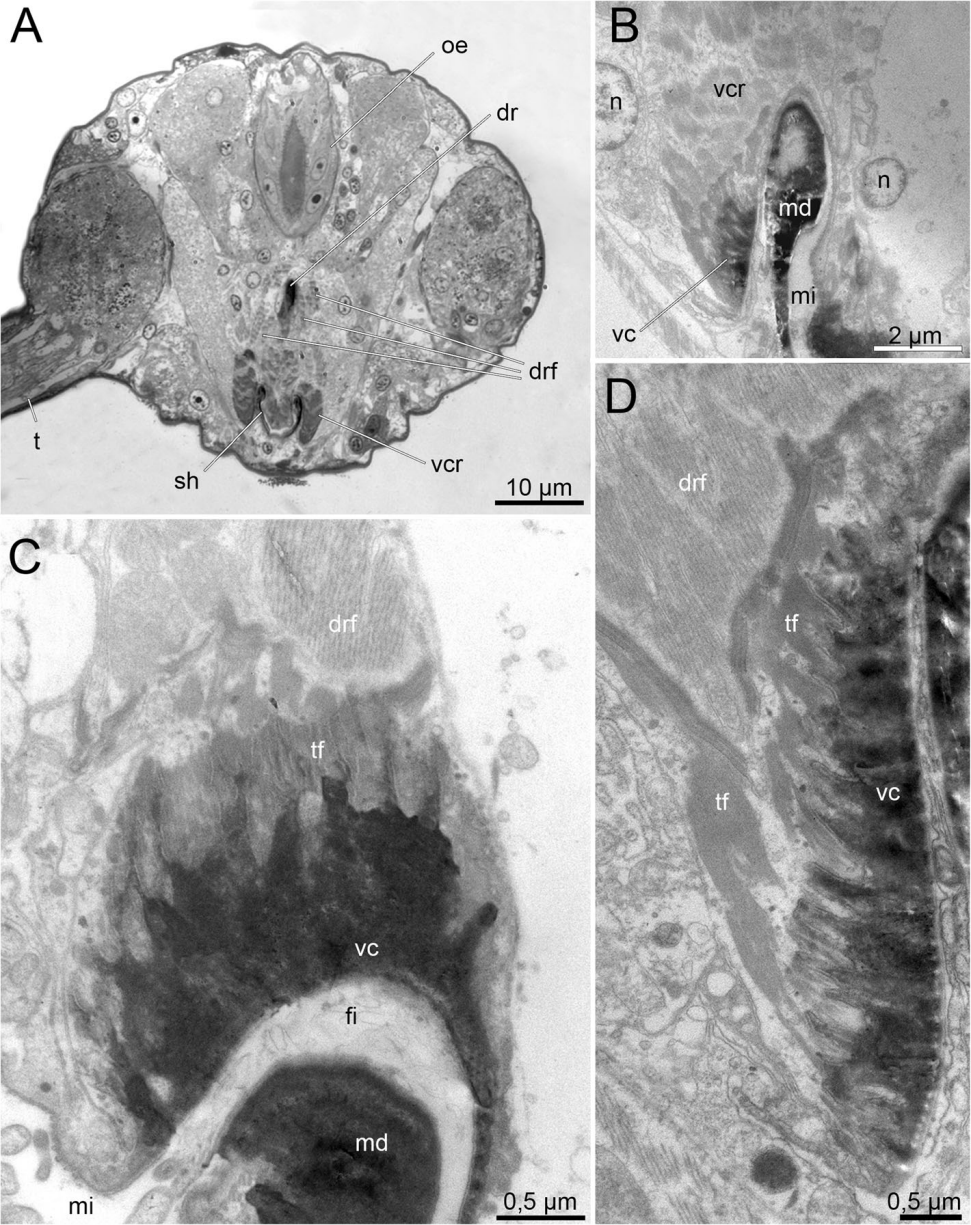
Fine structure of the ventral carrier and mandibular shaft of *Histriobdella homari*. Transversal section through the median region of the adult mandibular shaft. Light microscopy (**a**) and TEM images (**b**-**d**). **a**. Semithin section through the ventral pharyngeal organ. **b.** General view of the ventral carriers and left mandibular shafts. **c.** Fine structure of the ventral carrier and dorsal portion of the right mandibular shaft (note the structure between the ventral carrier and the mandible). **d**. Fine structure of the left ventral carrier. dr, dorsal rod; drf, dorsal rod flexor; fi, fibrous-like substance; md, mandible; mi, median VPO invagination; n, nucleus; oe, esophagus; sh, mandibular shaft; t, tentacle; tf, tonofilaments; vc, ventral carrier; vcr, ventral carrier retractor

In addition to the mandibles and maxillae, the histriobdellid jaw possesses additional supportive structures – the paired ventral carriers and the unpaired dorsal rod. The ventral carriers are represented by paired hardened structures with a complex (intricate) shape and a length of approximately 20 μm (Fig. 7b, c). They are attached to the maxillary apparatus at the bases of mxI and mxII and to the gnathoepithelium on the dorsal side (Figs. 7с; Fig. 10b-c). The massive lateral portions of the carriers cover the upper (bent to the dorsal side) edges of the mandibular shafts and slide along these shafts as if on rails. TEM microphotographs show the presence of a fibrous-like substance between the edges of the mandibular shafts and the carriers that probably serves as a lubricant (Fig. 10c). The cellular origin of this substance remains unknown and needs further investigation.

The unpaired dorsal rod, the second prominent structure supporting the mandibles and maxillae, has a length of 70–75 μm and is formed in a deep invagination of the gnathoepithelium (Fig. 4a; Fig. 5a; Fig. 6b; Fig. 10a). The paired basal (anterior) part of the dorsal rod is rooted in the maxillary complex, in the area of the fused basal parts of left and right mxI and mxII (Figs. 7b-c). The dorsal rod reaches back towards the posterior part of the VPO and ends at approximately the same level as the mandibular shaft (Fig. 7b). In histological sections, it is obvious that the dorsal rod is located in a deep invagination of the gnathoepithelium. Accordingly, it resembles a stick-like structure synthesized by the surrounding cells (Figs. 4a; Fig. 5a; Fig. 10a). The core of the rod consists of a dense matrix formed by merged electron-dense granules, similar to all other jaw elements described above (Fig. 6b). The size of these granules can be up to 0.02–0.04 μm. Three to four gnathoblast cells surrounding the dorsal rod are visible in cross-section along its median part (Figs. 5a; Fig. 6b; Fig. 10a).

The dorsal rod in its definitive shape and size is formed during early development in juveniles still inside the egg membrane (Fig. 7a). In adult *Histriobdella,* the gnathoblasts surrounding the dorsal rod no longer exhibit prominent microvilli. No distinct features supporting the synthetic activity of the respective cells can be identified. The gnathoblasts have elongated nuclei up to 5 μm in length and approximately 1 μm in thickness. Furthermore, tonofilaments are well developed and provide a tight connection of the dorsal rod with the dorsal rod flexors (Fig. 6b).

### Fine structure of the pharyngeal and epidermal epithelium

**Fig. 11:**
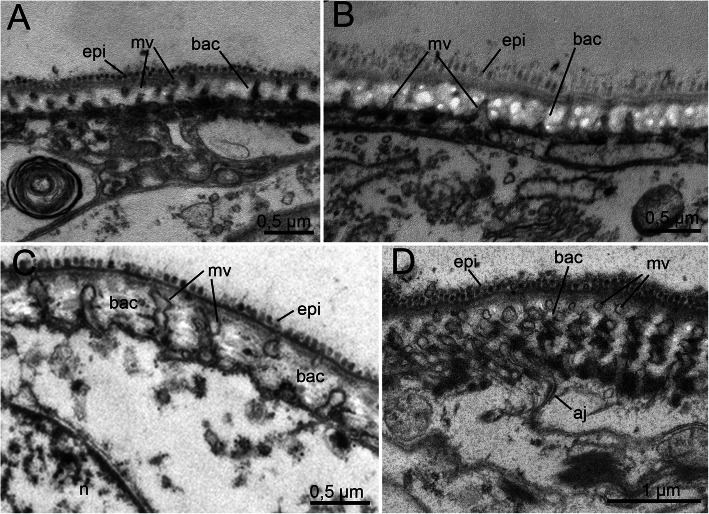
Fine structure of the juvenile (**a-b**) and adult (**c-d**) external epithelial cuticles of *Histriobdella homari*. TEM images. aj, adherens junction; bac, basicuticle; epi, epicuticle; mv, microvilli; n, nucleus

The ectodermal epithelium of the pharynx and epidermis consists of flattened cells approximately 3–4 μm in height, equipped with elongated nuclei 2 μm in diameter and approximately 3–5 μm long. The cuticle itself represents a “typical” annelid cuticle with a well-developed layer of epicuticle, dense microvilli and 3–4 layers of collagen fibers in the basicuticle (Figs. 11c-d). The thickness of the cuticle varies from 0.25 to 0.5 μm. Interestingly, juvenile specimens extracted from the egg envelope also show the adult-like set of cuticle features, with well-developed layers of collagen fibers (Fig. 11a-b).

Our investigations did not uncover significant differences in the pharyngeal epithelium between juveniles already possessing jaws (but remaining inside the egg envelope) and adult specimens. Both cell types, i.e., the pharyngeal epithelial cells as well as the gnathoblasts, are underlain by a thin basal lamina (approximately 0.1 μm) (Fig. 9; Fig. 11).

The pharyngeal epithelium is covered by the cuticle and has no distinct ciliation, except for the heavily ciliated epithelial cells of the esophagus (Fig. 1b; Fig. 9a-f; Fig. 10a). The cuticle of pharyngeal epithelial cells has a well-developed epicuticular layer of approximately 0.1 μm in thickness. The basal cuticular layer has almost the same thickness. The microvilli are quite dense but short (see Fig. 9d-e; Fig. 10f). The epithelial cells of the posterior-most part of the median invagination are approximately 2–3 μm in height, with elongated nuclei of 5 μm in length and approximately 1.5–2 μm in diameter. The cuticle of the epithelial cells covers the median invagination and appears as a thin layer of the electron-dense epicuticle (less than 0.1 μm). Notably, these epithelial cells exhibit almost no microvilli. Additionally, a basal cuticle layer is not visible in the TEM micrographs (Fig. 5a; Fig. 6b). The posterior-most part of the median invagination covers the dorsal side of the ventral muscle bulb. This part of the epithelium is represented by a very thin cellular layer (approximately 0.3–0.2 μm) with a very thin cuticle without any visible microvilli (Fig. 8c). In general, the epithelium of the VPO almost lacks glandular cells, while a few pairs of salivary glands with long ducts open on the dorsal side of the mouth cavity.

## Discussion

The combination of four different methods (i.e., electron microscopy, histological sectioning, 3D reconstruction, and immunohistochemical analyses) employed here allowed an in-depth description of the anatomical features of the *Histriobdella* jaw apparatus. In summary, our results largely corroborate the findings of [25] but also broaden the basis for comparison of the jaw apparatus in histriobdellids with that in other eunicidan families. According to our data, the anatomy of the ventral pharyngeal organ (VPO), despite the complexity and adaptive changes in its structure, fits the general pattern known for the VPO in other Eunicida (see scheme in Fig. 6). While the homology of maxillary dental plates and mandibles in histriobdellids and other eunicidans did not raise doubts, the homologies of the paired ventral carriers and unpaired dorsal rod with the jaw elements in other families were difficult to interpret [25]. Following [5], the supporting apparatus of the eunicid jaws consists of paired dorsal carriers and an unpaired ventral carrier, which has only been described for Oenonidae. Onuphidae and Eunicidae might also exhibit a structure similar to that of an unpaired ventral carrier [6].

Based on our investigations, the histriobdellid dorsal rod is histologically and positionally similar to the “carrier-like structure” found in Dorvilleidae [18] and in larval jaws of Onuphidae [12]. Accordingly, the dorsal rod is formed through deep invagination of the gnathoepithelium, which exhibits an unpaired jaw element. The putative function of this element seems to be the same as that of the other supporting elements of the Eunicida jaws – the anchoring of muscles, which are used to manipulate the jaw apparatus. However, in Histriobdellidae, the dorsal rod also acts as an antagonist of the retractors of the maxillary complex, while distinct protractor muscles are absent.

Despite the fact that the ventral carriers move along the mandibular shafts, they are part of the maxillary apparatus since they are connected to the bases of maxillae I and II. The gnathoepithelium producing the ventral carriers is located on the lateral sides of the median invagination, where the maxillary gnathoepithelial zone is located.

We suggest that the evolution of the unique paired ventral carriers is the result of the miniaturization of Histriobdellidae. A dramatic decrease in the number of VPO muscle cells led to extension of the spring-like dorsal rod to support the functionality of the entire structure. Simultaneously, the ventral carriers sliding along the shafts of the mandibles stabilize the feeding process and direct the movement of the whole maxillary apparatus. Interestingly, paired ventral carriers appear to be an autapomorphy of Histriobdellidae and have not been observed in any other extant Eunicida – at least not on the same level of structural complexity. Such paired structures are also unknown in the fossil record. Notably, in the process of stomodeum organogenesis, the ventral carriers appear latest of all elements of the maxillary apparatus.

All other components of the maxillary apparatus – four pairs of maxillae and an unpaired supporting element represented by the dorsal rod – resemble the ctenognath jaw apparatus characteristic of Dorvilleidae [20] (Fig. 7, Table 1). Accordingly, the ultrastructure and position of the dorsal rod in histriobdellids are highly comparable to those of the carrier-like structures known from other ctenognath jaws [6, 20]. Additionally, the histriobdellid maxillary apparatus does not exhibit dorsal paired carriers, which are shown to be a main characteristic of prionognath jaws (39, see also Table 1).

Furthermore, our investigations indicate that a significant part of the jaw apparatus – the maxillae II, mandibles and the dorsal rod – are fully developed in juveniles that have not yet emerged from the egg envelope. The remaining components of the jaw apparatus, namely, maxillae I, III and IV and the ventral carriers, develop shortly after juveniles hatch from the egg, since all free-living individuals (even the smallest examined juveniles) had a definitive and fully developed adult-like jaw apparatus. Such presence of an adult-sized jaw apparatus in juveniles hints at a pedomorphic developmental mode for the mentioned structure in histriobdellids. Whether pedomorphosis is responsible for the observed developmental characteristics must be investigated in the future, but comparable processes are well documented for several annelid families [32–34].

To provide an in-depth comparative analysis of recent and fossil jaws in different families of Eunicida, more investigations dealing with the fine structure and development of the jaw apparatus are needed. This information is currently unavailable for two major eunicidan families, namely, Lumbrineridae and Oenonidae. For other eunicid groups, detailed investigations are also quite limited [6].

**Fig. 12:**
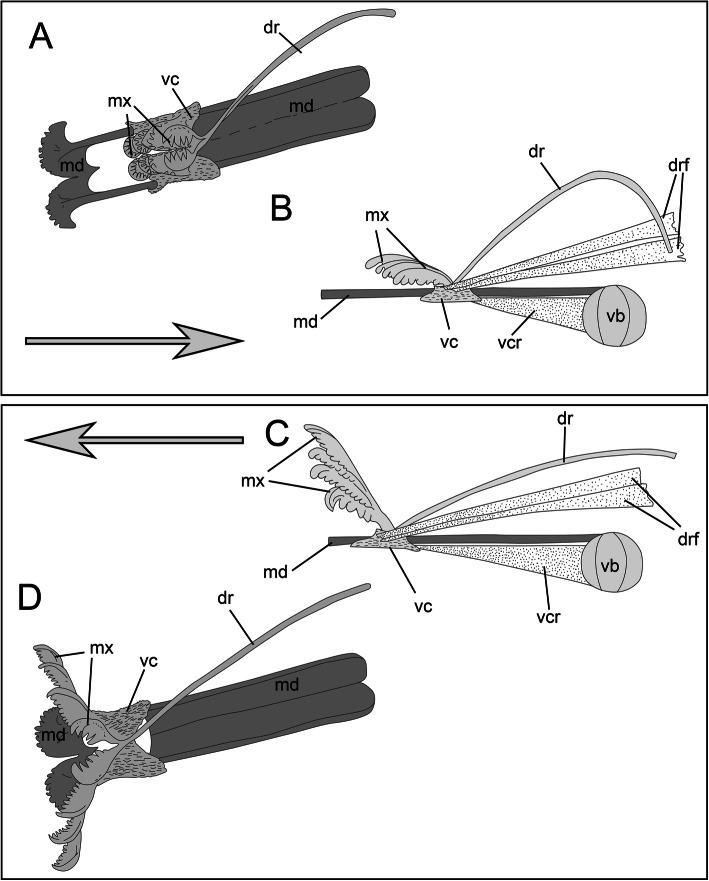
Scheme of the jaw movement of *Histriobdella homari*. Putative function of the dorsal rod and associated muscular elements of the VPO during the retraction (**a**, **b**) and protraction (**c**, **d**) of the maxillae. The arrows indicate the direction of movement. dr, dorsal rod; drf, dorsal rod flexor; md, mandible; md, mandibles; mx, maxillae; vb, ventral muscular bulb; vc, ventral carriers; vcr, ventral carrier retractor

In regard to the mechanical principles of the histriobdellid jaw apparatus, our results confirm and supplement the data of Jennings & Gelder [25]. Thus, the ventral carrier retractors can move the entire maxillary apparatus backwards relative to the front edge of the mandibles, while the flexor muscles of the dorsal rod bend this element into an arc. As a result, the maxillary complex moves even farther backward, and the entire maxillary apparatus folds and moves inward to the median invagination (see also Fig. 12 a-b). Subsequently, when the ventral carrier retractors (two cells) are relaxed, the ventral carriers and the entire maxillary complex can move forward. This movement is also possible due to the extension of the dorsal rod, which functions like a spring. The pressure of the front end of the dorsal rod not only leads to the advancement of the maxillary apparatus in the forward direction but also causes the closure of the maxillae (Figs. 12c-d). Thus, the movement of the maxillae together with the frontal edge of the mandibles pressed against the substrate allows scraping bacterial fouling from the surface of the lobster gills and eggs.

The muscular apparatus of other small and putatively miniaturized annelids, such as Nerillidae, Diurodrilidae, Dinophilidae and interstitial Dorvilleidae, usually possesses a meshwork of numerous muscle fibers covering the VPO in high complexity [35–38]. In contrast, the number of muscle cells in *Histriobdella*’s VPO is rather small and strongly constant. The whole muscular apparatus of the VPO consists of only three muscle cells of the bulb, one pair of retractor cells of the carriers and a total of 18 cells (left and right) of flexors of the dorsal jaw apparatus, findings supporting those of earlier investigations by Jennings and Gelder [25]. In *Histriobdella,* the VPO muscle cells do not form a continuous muscle layer, as in most other polychaetes with a VPO (investing muscles, [13]). Instead, the muscles are present as individual muscle elements, which are not covered with a coelomic epithelium. Quite comparable conditions have been described only for Dinophilidae, another miniature group of annelids [35, 36]. Nevertheless, detailed comparisons reveal differences between the taxa, such as the number of involved muscle fibers and the orientation of the muscular complex. Accordingly, in Dinophilidae, the pharyngeal bulb consists of 27–28 plate-shaped muscle cells, and the bulbus muscles are connected to six longitudinal muscle cells on their dorsal side. Furthermore, the entire pharyngeal bulb complex in dinophilids comprises two sets of longitudinal muscle systems – three small cells in the ventral region and 11–15 pairs of dorsal longitudinal muscle cells connecting the dorsal parts of the bulb with the pharyngeal fold. Such an assemblage drastically differs from the histriobdellid morphology. Hence, the simplification of the muscular scaffold surrounding the VPO and the jaws might have evolved convergently in both lineages due to miniaturization. Further analyses – also at the molecular level – are needed to test this hypothesis.

The ultrastructural data on the fine morphology of the *H. homari* stomodeum provide further support that the jaws of these animals are very similar to the jaws of Dorvilleidae [13, 18], as well as to the larval jaws of Onuphidae [12]. In all three cases, the jaw plates are formed via thickening of the cuticle and impregnation of its outer layer, with dark electron-dense granules merging into solid, dense plates. Interestingly, the formation of jaw plates in the sedentary ampharetid *Adercodon pleijeli* [13, 39] also occurs due to the secretion and fusion of granules of the same size and secretion of scleroproteins in the epicuticle zone. As these substances accumulate, the epicuticle becomes thicker, and electron-dense spheroid granules merge into a monolithic mass – the jaw plate.

In *Histriobdella,* the gnathoepithelial cells forming the jaw elements underlie the jaw plates along their inner surface. They are actively functioning cells equipped with mitochondria and a distinct synthetic cellular apparatus in the basal zone. In contrast, the gnathoepithelium in the distal parts of the jaw plates is represented by somewhat degenerated parts of cells lacking mitochondria and endoplasmic reticulum but filled with an unstructured gray mass. This is very similar to the state of gnathoepithelial cells in the distal maxillary parts in Dorvilleidae [13, 18] and larval jaws of Onuphidae [12]. Our data suggest that after formation of a jaw plate, the cells of the gnathoepithelium retain only a small number of microvilli or lose them completely. Such jaws do not have the potential for long-term growth and thickening, and once formed, they remain unchanged throughout the life of an animal (Histriobdellidae) or are shed over time while the new jaws arise from new sites of the gnathoepithelium (jaws of Dorvilleidae or larval jaws of Onuphidae, [13, 18, 19]). Nevertheless, the epithelium of the body surface is a typical annelid monolayered ectodermal epithelium with a well-developed basal lamina. The cuticle of the epidermis shows all characters of a typical annelid cuticle, with an epicuticle, a basicuticle, and a regular multilayered arrangement of collagen fibers in the basal cuticle layer penetrated by microvilli [40].

The stomodeum epithelium does not bear cilia, while the ciliature of the esophageal epithelium is very well pronounced – a condition that is similar to that in Dorvilleidae and Onuphidae as well [12, 13, 18]. The cuticle of the stomodeum epithelium is a so-called “larval-type” cuticle [39]. Hence, collagen fibers of the basicuticle are arranged amorphously, without distinct layers, and are almost nonvisible in the TEM micrographs.

## Conclusions

Our comparative and comprehensive investigations using a set of different morphological methods highly support the phylogenetic placement of Histriobdellidae within the Eunicida.

The quite simplified muscular scaffold surrounding the histriobdellid jaw apparatus might be the result of body miniaturization and adaptive evolution. Nevertheless, the ultrastructural characteristics of the histriobdellid jaws are very similar to those of Dorvilleidae jaws [11] and the larval jaws of *Mooreonuphis stigmatis* (Onuphidae) [12] in terms of cell shape and size within the gnathoepithelium and the arrangement of granules within the jaw-forming epithelia. Unfortunately, data for adult onuphids are scarce; therefore, only comparisons to larval characteristics are possible. Furthermore, our data indicate that the maxillae of *Histriobdella homari* are of the ctenognath jaw type, with an unpaired dorsal carrier-like element (dorsal rod), unique ventral carriers and the lack of prionognath-like dorsal carriers. Comb-shaped ctenognath jaws have also been described in Dorvilleidae [[12], Table 1]. In summary, our dataset supports a close relationship between Histriobdellidae and Dorvilleidae based on the anatomy and ultrastructure of the jaw apparatus. Nevertheless, further analyses including additional datasets such as molecular markers are needed to verify this hypothesis.

## Data Availability

For data transparency, all aligned semithin sections are freely available from the Zenodo data repository (https://zenodo.org/) and can be found using the following DOIs: - *Histriobdella homari*, semithin serial sections, 50x magnification: 10.5281/zenodo.4047732 - *Histriobdella homari*, semithin serial sections, 100x magnification: 10.5281/zenodo.4047746
